# Clinical and economic impact of universal varicella vaccination in Norway: A modeling study

**DOI:** 10.1371/journal.pone.0254080

**Published:** 2021-07-08

**Authors:** Manjiri Pawaskar, Colleen Burgess, Mathew Pillsbury, Torbjørn Wisløff, Elmira Flem

**Affiliations:** 1 Center for Observational and Real-World Evidence (CORE), Merck & Co., Inc., Kenilworth, NJ, United States of America; 2 Department of Community Medicine, UiT The Arctic University of Norway, Tromsø, Norway; 3 MSD, Drammen, Norway; Texas A&M University College Station, UNITED STATES

## Abstract

**Background:**

Norway has not implemented universal varicella vaccination, despite the considerable clinical and economic burden of varicella disease.

**Methods:**

An existing dynamic transmission model of varicella infection was calibrated to age-specific seroprevalence rates in Norway. Six two-dose vaccination strategies were considered, consisting of combinations of two formulations each of a monovalent varicella vaccine (Varivax^®^ or Varilrix^®^) and a quadrivalent vaccine against measles-mumps-rubella-varicella (ProQuad^®^ or PriorixTetra^®^), with the first dose given with a monovalent vaccine at age 15 months, and the second dose with either a monovalent or quadrivalent vaccine at either 18 months, 7 or 11 years. Costs were considered from the perspectives of both the health care system and society. Quality-adjusted life-years saved and incremental cost-effectiveness ratios relative to no vaccination were calculated. A one-way sensitivity analysis was conducted to assess the impact of vaccine efficacy, price, the costs of a lost workday and of inpatient and outpatient care, vaccination coverage, and discount rate.

**Results:**

In the absence of varicella vaccination, the annual incidence of natural varicella is estimated to be 1,359 per 100,000 population, and the cumulative numbers of varicella outpatient cases, hospitalizations, and deaths over 50 years are projected to be 1.81 million, 10,161, and 61, respectively. Universal varicella vaccination is projected to reduce the natural varicella incidence rate to 48–59 per 100,000 population, depending on the vaccination strategy, and to reduce varicella outpatient cases, hospitalizations, and deaths by 75–85%, 67–79%, and 75–79%, respectively. All strategies were cost-saving, with the most cost-saving as two doses of Varivax^®^ at 15 months and 7 years (payer perspective) and two doses of Varivax^®^ at 15 months and 18 months (societal perspective).

**Conclusions:**

All modeled two-dose varicella vaccination strategies are projected to lead to substantial reductions in varicella disease and to be cost saving compared to no vaccination in Norway.

## Introduction

Varicella, or chickenpox, is caused by the varicella zoster virus and is one of the most common infectious diseases in children. Varicella infections are characterized by a generalized pruritic vesicular rash, which, while usually mild, can result in serious complications and, in rare cases, death [1]. Almost all people in Norway eventually contract varicella, with a seroprevalence in adults ≥45 years of 95% [2]. Herpes zoster, or shingles, a second clinical manifestation of the varicella virus, may occur later in adulthood upon reactivation of latent virus in nerve ganglions [1].

Varicella vaccines are well-tolerated and effective in preventing varicella. Where introduced, universal childhood vaccination has led to significant declines in varicella disease [3–7]. Globally, two-dose varicella vaccination programs have an average effectiveness of 92% [4]. For example, in Germany, where childhood vaccination against varicella was introduced as a one-dose schedule in 2004 and a two-dose schedule in 2009, varicella incidence declined by 55% among all age groups between April 2005 and March 2009 and by 63% in the 0–4 year old age group during that same period [8]. Furthermore, in the United States, where universal childhood vaccination against varicella was introduced as a one-dose schedule in 1996 and a two-dose schedule in 2006, varicella incidence declined by 90% between 1995 and 2008 [6].

Varicella vaccination is recommended only for selected risk groups in Norway [9] and is not included in the childhood immunization program [10]. In the absence of universal vaccination, varicella causes a substantial healthcare burden in Norway [11]. During 2008–2014 the primary healthcare incidence of varicella was 221 per 100,000 population with most cases occurring in children less than 5 years of age [11]. Concurrently, there was a varicella hospitalization rate of 7.3 cases per 100,000 population and a varicella death rate of 0.6 per million population [11]. The estimated annual varicella-specific healthcare costs in 2017 were Norwegian Kroner (NOK) 23 million [12].

To support national vaccination policy decisions, the study objective is to quantify the long term clinical and economic impact of universal varicella vaccination in Norway under different vaccination scenarios using a dynamic transmission model.

## Materials and methods

### Dynamic transmission model

The deterministic, compartmental model of varicella infection used for this analysis has been described in detail elsewhere [13]. It is a mathematical description of the natural history and treatment of varicella disease in a population stratified by age, and is an adaptation of models employed by other investigators [14–17]. Calibrated to the demographic, behavioral, and epidemiological characteristics of the population of Norway, the model estimates the population-level impact of varicella vaccination over a time horizon of up to 100 years (the base case is 50 years). The model computes the impact of varicella vaccination on varicella and herpes zoster incidence rates, and on varicella-related mortality, healthcare use, and direct and indirect costs. Model inputs are provided in S1 Appendix.

#### Model components

The model has several components: demographic, epidemiologic, vaccination, and economic. The demographic component of the model defines the age structure of the population simulated and describes how persons enter, age, and exit the model [18]. Demographic input variables—total population, fertility rate, and all-cause mortality—by 5-year age groups were derived from the United Nations World Population Prospects database files, 2019 revision [19]. The epidemiological component of the model simulates the dynamics of varicella infection. Periods of passive immunity, latency, and infectiousness were as reported elsewhere [13]. Briefly, the average passive immunity was 6 months, the average latent period (natural and breakthrough [defined as varicella disease occurring >42 days after vaccination] varicella) was 14 days, and the average infectious period for natural varicella was 7 days. Breakthrough varicella was assumed to be 50% as infectious as natural varicella with a 20% mortality rate [16, 20], and the average infectious period of breakthrough varicella was assumed to be 4.5 days.

Also, as in the previous work, we incorporated the effects of exogenous boosting on herpes zoster incidence [21], and the calibration of herpes zoster reactivation rate included this assumption. Our assumptions about exogenous boosting were based upon the temporary immunity approach presented in Wolfson et al., 2019 [13]. Assumptions used for force of boosting and duration of protection are consistent with prior studies [20, 22]. Our model assumed that persons who have low immunity to HZ are boosted when they come into contact with infectious persons at the same rate as susceptible persons become exposed with varicella, which is a higher rate of boosting than that used in other models [20, 22, 23]. The duration of protection against HZ was assumed to be 80 years in this model, which is comparable to the duration of protection provided by two doses of varicella vaccine based upon performance parameterization [20, 22]. This is also consistent with modeling studies that assumed lifelong protection [23]. The model was not adjusted for age due to parsimony, an approach we share with other models [24].

**Fig 1:**
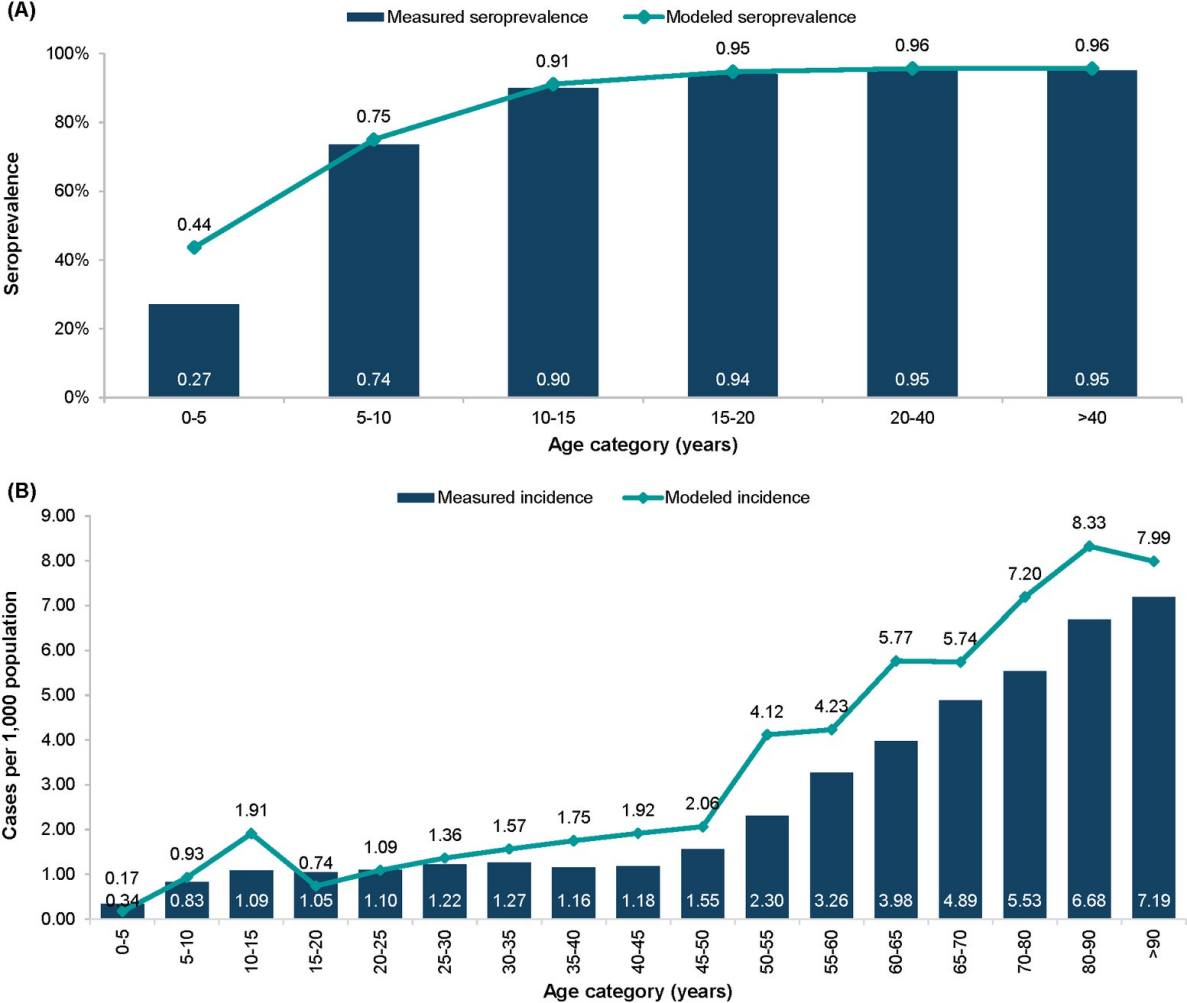
Model fit to (A) varicella seroprevalence and (B) herpes zoster incidence rates in Norway.

The model is calibrated to the population of Norway using population-specific initialization parameters, including the age distribution of the population, age-specific fertility, varicella seroprevalence (in the absence of vaccination), and herpes zoster incidence by age. Age-specific incidence rates of varicella were obtained by finding a best fit to the seroprevalence rates [25]. The age-specific incidence of herpes zoster was simulated by fitting modeled rates to the observed incidence rates [11]. The comparisons of the observed and model-simulated varicella seroprevalence and herpes zoster incidence are presented in Fig 1.

The varicella vaccination component of the model implements a structure similar to that described elsewhere [17]. Vaccine parameters were: ‘failure’, the proportion of vaccinations that confer no varicella immunity; ‘take’, the initial seroconversion; ‘degree of protection’, the proportion of individuals fully protected from breakthrough disease; and ‘duration of immunity’, the rate at which individuals transition from full to partial protection against breakthrough disease [26].

The economic component of the model calculates healthcare usage and costs associated with varicella infection, with costs expressed in NOK (2020 value). The time horizon was 50 years [27]. Direct costs were computed as costs borne by the healthcare system. Indirect costs—the costs of lost workdays due to varicella infection—were included in costs from the societal perspective. The indirect cost calculations within the model also accommodate cost of workdays lost as well as travel costs for vaccination, however the latter were assumed to be negligible for Norway, and thus did not impact indirect costs. Vaccination cost included the list price of the vaccine and the administration cost, estimated as the cost of 10 minutes at a nurse’s salary of 436 NOK per hour [28]. Indirect costs were based on current infection control guidelines for control of varicella outbreaks and the mean wage for Norway, as reported by Statistics Norway [29]. Costs were discounted by 3%. Rates of primary healthcare visits, hospitalizations, and mortality associated with varicella infection, and unit costs, were as reported elsewhere [11, 12]. Varicella morbidity and mortality outcomes were aggregated to estimate quality-adjusted life years (QALYs), a generic measure of disease burden that indicates both the quality and quantity of life years lived. Utility values used to calculate QALYs were as reported elsewhere [13, 30]. Both costs and QALYs were discounted at a rate of 3% per year.

### Vaccination strategies

Six two-dose vaccination strategies were modeled, labelled A through F, with the first dose at 15 months and the second dose at 18 months, 7 years, or 11 years (Table 1). The ages for vaccine administration were chosen to match the existing childhood vaccination program in Norway, which includes immunization against measles-mumps-rubella (MMR) at 15 months, immunization against diphtheria, tetanus, pertussis, and poliomyelitis (DTP-IPV) at about 7 years, and a second immunization against MMR at age 11–12 years [10]. Although no vaccines are currently administered at 18 months of age, there is a group consultation at this age, which may be converted to an immunization visit. The first dose was assumed to be administered as a monovalent varicella vaccine. The second dose was assumed to be administered either as a monovalent vaccine, or as a quadrivalent measles-mumps-rubella-varicella (MMRV) vaccine. All currently available varicella-containing vaccines—the monovalent vaccines Varivax® (Merck & Co., Inc., Kenilworth, NJ, USA, V-MSD) and Varilrix® (GlaxoSmithKline, V-GSK), and the quadrivalent vaccines, ProQuad® (Merck & Co., Inc., Kenilworth, NJ, USA, MMRV-MSD) and Priorix-Tetra® (GlaxoSmithKline, MMRV-GSK)—were included. Vaccine parameters for V-MSD and MMRV-MSD were as reported elsewhere [17, 31]. Vaccine parameters for V-GSK and MMRV-GSK were based on clinical trial data [32], as described in modelling studies [33–35]. The monovalent and quadrivalent vaccines from the same manufacturer contain the same varicella strain and are considered to be immunologically equivalent [36].

**Table 1:** Vaccination strategies ^A^.

Strategy	Formulation		Age at Vaccination	
	1^st^ Dose	2^nd^ Dose	1^st^ Dose	2^nd^ Dose
A	Varivax®	Varivax®	15 months	7 years
B	Varilrix®	Varilrix®	15 months	7 years
C	Varivax®	ProQuad®	15 months	11 years
D	Varilrix®	Priorix-Tetra®	15 months	11 years
E	Varivax®	Varivax®	15 months	18 months
F	Varilrix®	Varilrix®	15 months	18 months

^A^ The base case is a time horizon of 50 years.

### Sensitivity analyses

A one-way sensitivity analysis was conducted from the societal perspective, in which variations in economic parameters and in parameters related to vaccine properties were analyzed. In the one-way sensitivity analysis, vaccine price and the costs of a lost workday, and of outpatient and inpatient care, were varied by ± 20%. Vaccination coverage for doses 1 and 2 were varied by ± 5% (vaccination coverage of 2-year-olds in Norway is 95% to 97%; coverage with measles, poliomyelitis, and diphtheria-tetanus-whooping cough vaccines in 16-year-olds is 94% to 95% [37]). Vaccine efficacy for Varivax® was varied by 20% (bounded at the top by 100%) [31], and for Varilrix® by ± 5% [38]. Time horizons of 25 and 100 years and 4% discounting were also considered. Additional scenario analysis was conducted to assess the impact of including the direct and indirect cost of herpes zoster (details are provided in Tables 2 and 3 in S2 Appendix) on QALYs and the cost-effectiveness of interventions. Probabilistic sensitivity analysis was conducted as described elsewhere [13]. Five hundred sets of parameter value variations were computed using Latin Hypercube Sampling for each vaccination strategy.

## Results

### Varicella incidence by vaccination strategy

In the absence of varicella vaccination, the projected annual incidences of natural varicella and wild-type herpes zoster in Norway are 1,359.4 per 100,000 population and 303.8 per 100,000 population, respectively.

**Fig 2:**
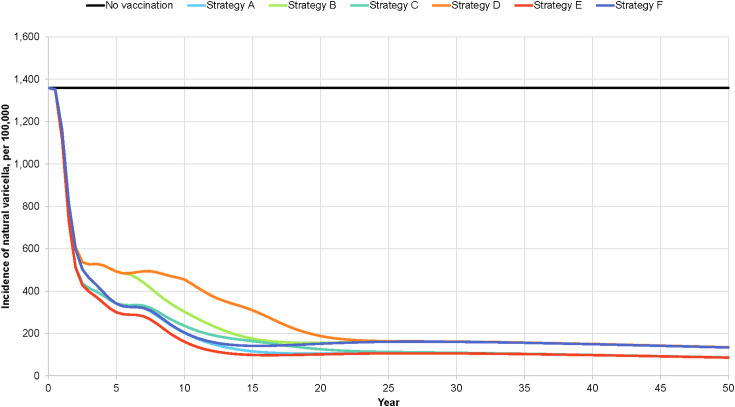
Projected incidence of total varicella, by varicella vaccination strategy.

**Fig 3:**
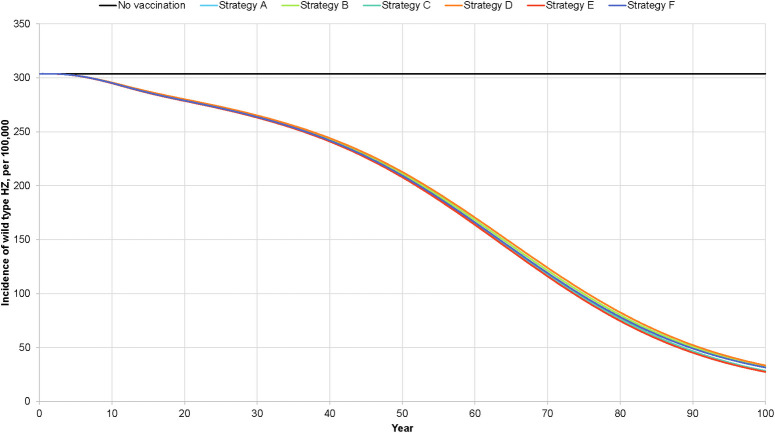
Projected impact of varicella vaccination on herpes zoster incidence.

With universal vaccination, depending on the vaccination strategy, the natural varicella incidence rate at 50 years is projected to vary from 48.4 to 58.9 cases per 100,000 population (Fig 2) and the breakthrough varicella incidence rate from 37.8 to 75.9 per 100,000 population. The wild-type herpes zoster incidence rate is projected to vary from 207.8 to 212.6 per 100,000 population (Fig 3). For each of these incidence rates, strategies E and D yielded the lowest and highest values, respectively. The projected decline in natural varicella incidence after the introduction of universal varicella vaccination is rapid, with a >50% reduction occurring within two years after the program start. Conversely, the projected decline in herpes zoster incidence is slow, with a 50% reduction occurring 62.5 to 65 years after the introduction of varicella vaccination.

### Outpatient cases, hospitalizations, and deaths

**Fig 4:**
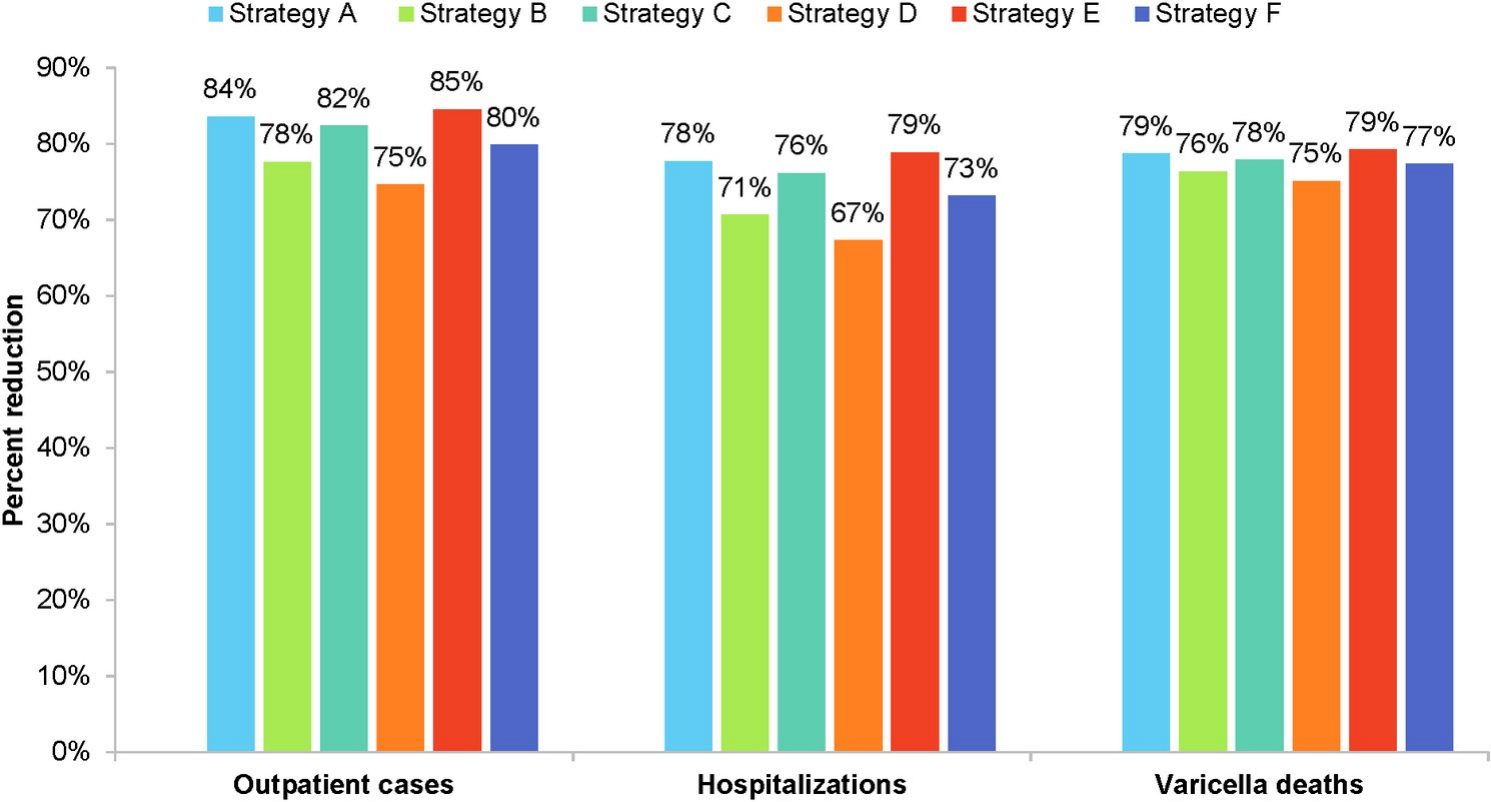
Projected impact of universal varicella vaccination on varicella primary care cases, hospitalizations, and deaths.

In the absence of vaccination, the cumulative numbers of varicella outpatient cases, hospitalizations, and deaths over 50 years are projected to be 1.81 million, 10,161, and 61, respectively. At 50 years, universal vaccination is projected to have reduced outpatient cases by 75%-85%, hospitalizations by 67%-79%, and deaths by 75%-79%. Strategies D and E result, respectively, in the lowest and highest percent reductions in outpatient cases, hospitalizations, and deaths (Fig 4).

### Cost-effectiveness

**Fig 5:**
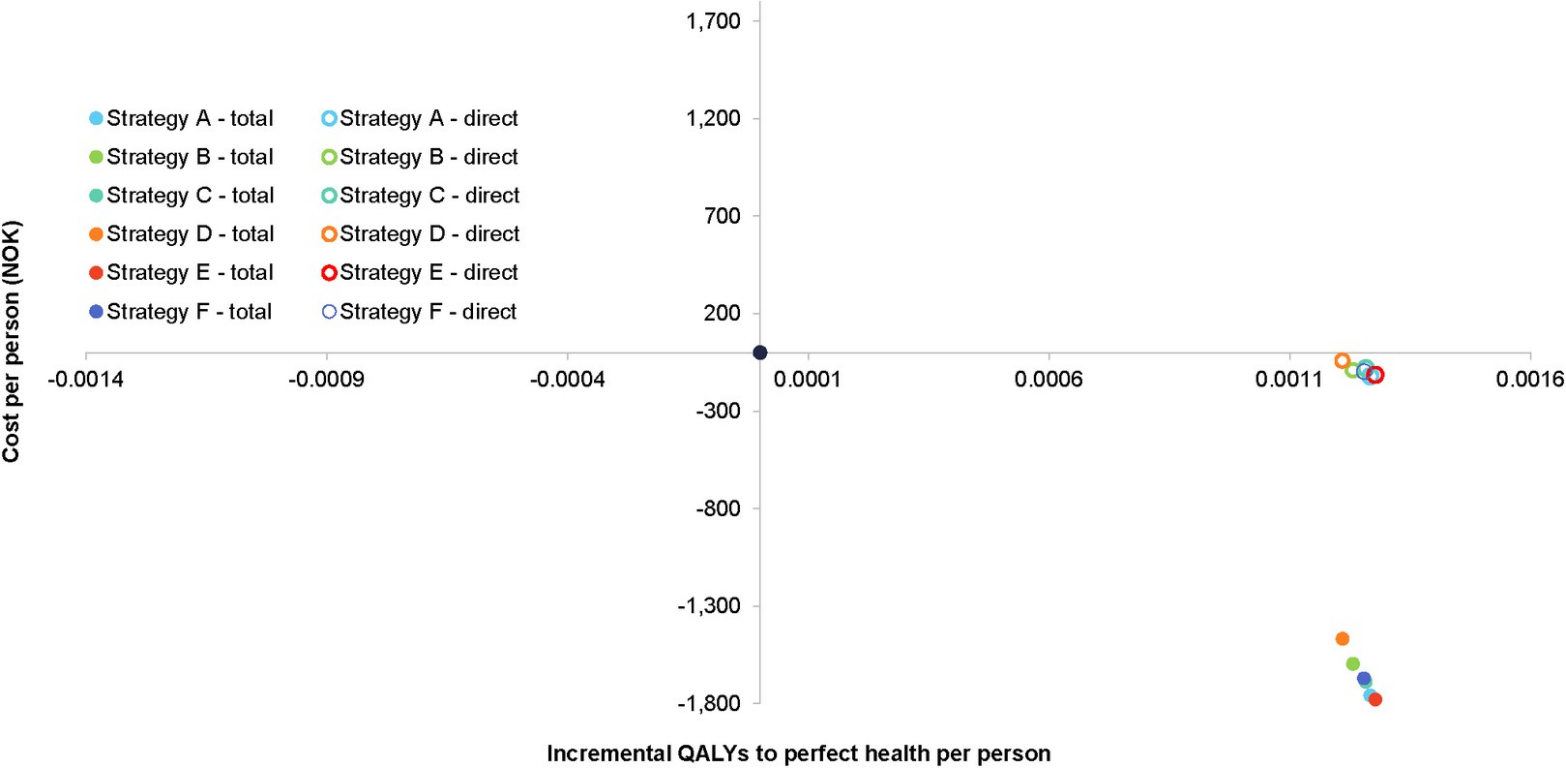
Cost-effectiveness of varicella vaccination from the health care system- and societal perspectives. Cost-effectiveness analysis from the perspectives of society (closed circles) and the health care system (open circles). Closed black circle represents no vaccine scenario.

The cost-effectiveness of the six varicella vaccination strategies compared with no vaccination is graphed in Fig 5. All cost-effectiveness points, from the perspectives of both the healthcare system and society, lie in the southeast quadrant of the cost-effectiveness plane, indicating dominance over no vaccination. From the perspective of the healthcare system, strategies A and E have the greatest reductions in costs over a 50-year time horizon: 120.41 and 113.67 NOK, respectively (Table 2). From the societal perspective, strategies E and A have the greatest cost reductions over a 50-year time horizon: 1,779 and 1,758 NOK, respectively (Table 3). Strategies E and A also had the greatest number of QALYs gained (0.00128 and 0.00127, respectively).

**Table 2:** Cost-effectiveness of varicella vaccination from the perspective of the health care system ^A^.

Vaccination strategy	Cost savings in NOK ^B^	QALYs gained	ICER ^C^
A	(120.41)	0.00127	Dominant
B	(90.61)	0.00123	Dominant
C	(79.91)	0.00126	Dominant
D	(40.94)	0.00121	Dominant
E	(113.67)	0.00128	Dominant
F	(96.36)	0.00125	Dominant

ICER, incremental cost-effectiveness ratio; QALY, quality-adjusted life years

^A^ The base case is a time horizon of 50 years.

^B^ Costs in NOK 2020.

^C^ All strategies result in cost savings and quality-adjusted life years gained over no vaccination.

**Table 3:** Cost-effectiveness of varicella vaccination from the societal perspective.

Vaccination strategy	Cost savings in NOK ^A^	QALYs gained	ICER ^B^
A	(1,757.62)	0.00127	Dominant
B	(1,595.62)	0.00123	Dominant
C	(1,687.85)	0.00126	Dominant
D	(1,467.24)	0.00121	Dominant
E	(1,778.50)	0.00128	Dominant
F	(1,670.31)	0.00125	Dominant

ICER, incremental cost-effectiveness ratio; QALY, quality-adjusted life years

^A^ Costs in NOK 2020. The parentheses indicate negative values which represent cost savings.

^B^ All strategies result in cost savings and quality-adjusted life years gained over no vaccination.

### Sensitivity analyses

**Fig 6:**
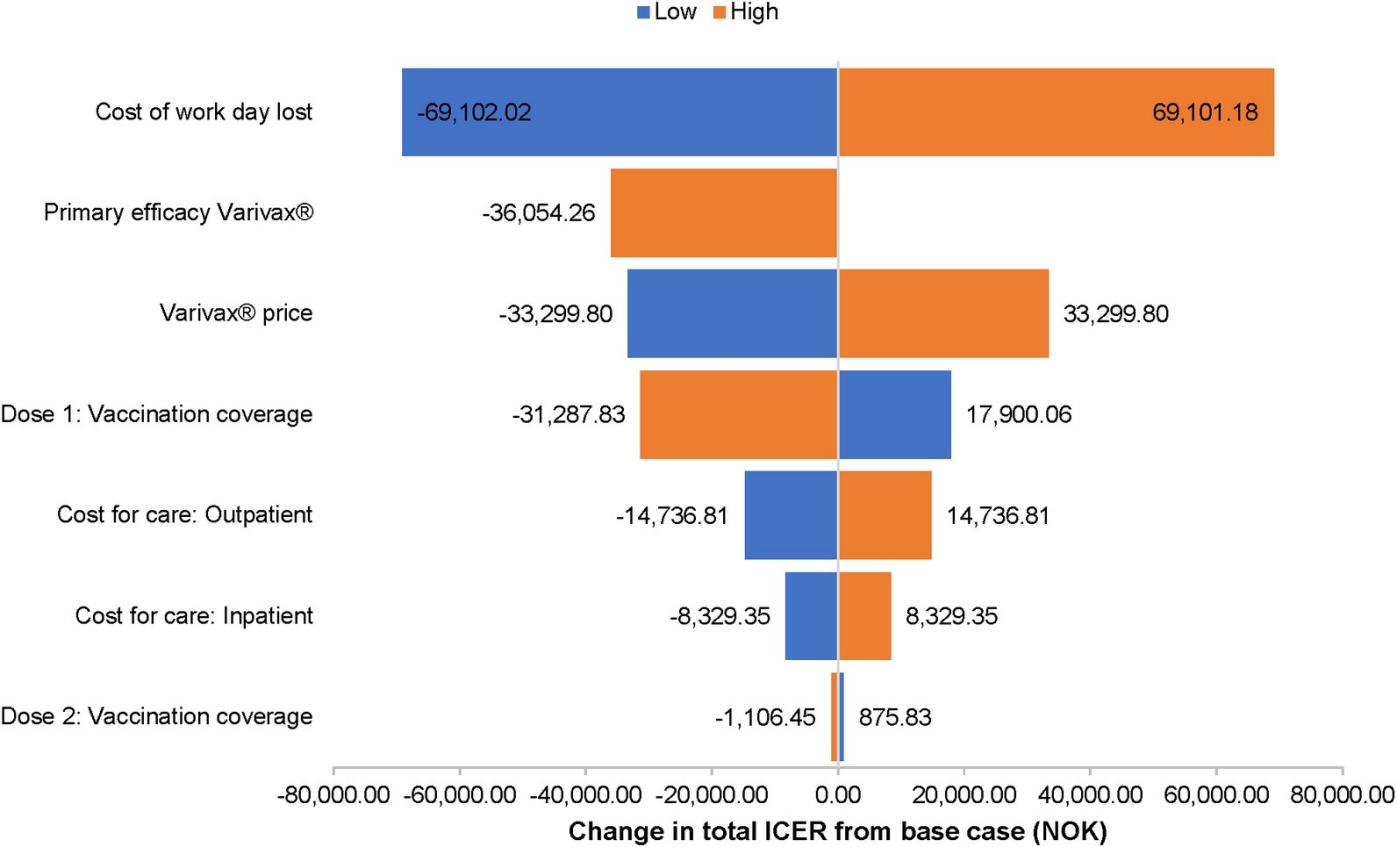
One-way sensitivity analysis of varicella vaccination strategy A compared to no vaccine. Sensitivity analysis from the societal perspective.

**Fig 7:**
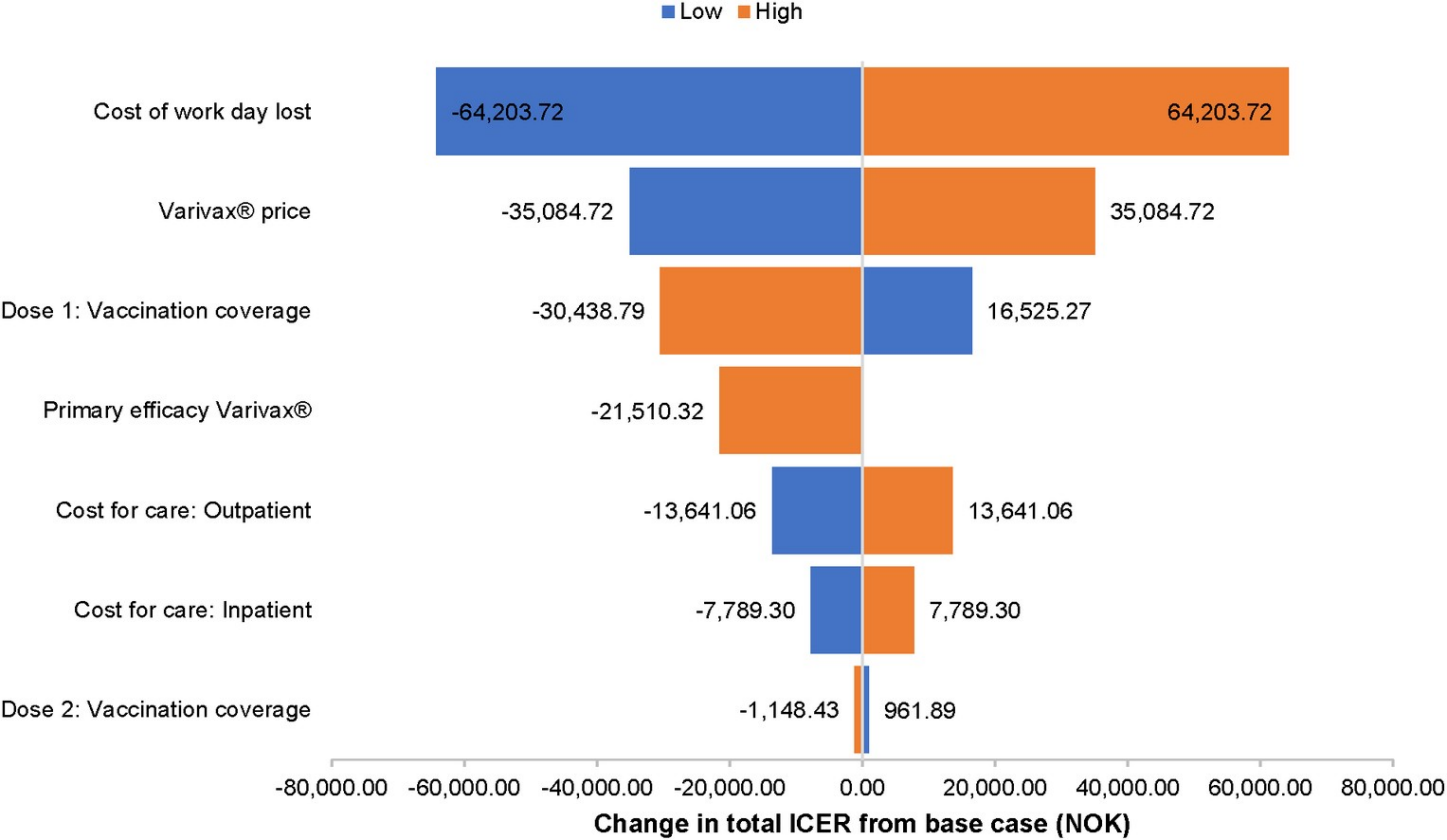
One-way sensitivity analysis of varicella vaccination strategy E compared to no vaccine. Sensitivity analysis from the societal perspective.

In the one-way sensitivity analysis of strategy A (Fig 6), the cost of a lost workday was the most important variable, changing the incremental cost-effectiveness ratio (ICER) by ± 69,100 NOK but demonstrating that the vaccine program would remain cost saving. The ICER is used to summarize the cost-effectiveness of a health care intervention. The ICER was also sensitive to vaccine take, vaccine price and coverage of the first vaccine dose. The one-way sensitivity analysis of strategy E produced similar results (Fig 7). The ICER was most sensitive to the cost of a lost workday, which changed the ICER by ± 64,200 NOK per QALY gained (still cost saving). Varying vaccine efficacy for first dose vaccination by -20% resulted in a +2.6% change in the ICER for strategy A from the societal perspective ICERs at the time horizon of 25 years were <3.0% lower than ICERs at 50 years, while ICERs at 100 years were less than 2% higher (Table 1 in S2 Appendix). Relative to the base case discount rate of 3%, applying 4% discounting changed the ICER by less than ± 0.4% at 25 years, less than + 0.5% at 50 years, and less than + 1% at 100 years (Table 1 in S2 Appendix). When the costs of of medically attended herpes zosterwere included in the cost-effectiveness analyses, the results were consistent with base case: all strategies were still cost-saving compared to no vaccination from both the health care system (Table 2 in S2 Appendix) and societal perspective (Table 3 in S2 Appendix).

## Discussion

In this study, all modeled two-dose vaccination strategies are projected to reduce natural varicella incidence by 95% to 96% relative to no vaccination in Norway. At the 50-year time horizon, all strategies are projected to reduce outpatient cases and hospitalizations by 67% to 85%, and deaths by 75% to 79%. A varicella vaccination program in Norway under all modelled strategies would be cost saving compared to no vaccination from the perspectives of both the healthcare system and society. From the societal perspective, most of the projected cost savings are due to reductions in lost workdays. Among the vaccination strategies, those with two doses of V-MSD, with the second dose at 18 months or 7 years (strategies E and A, respectively), were the most cost saving from the perspectives of both the healthcare system and society. The least impactful strategy in terms of cost-effectiveness and resource utilization was strategy D (V-GSK at 15 months and MMRV-GSK at 11 years).

These results are consistent with applications of the same dynamic transmission model to other countries [13, 27, 39]. Applied to Turkey, two-dose strategies of universal childhood varicella vaccination were projected to reduce varicella incidence by 99% after 25 years and to be cost-effective [13]. Two-dose vaccination strategies in Italy were projected to reduce varicella cases by 52% to 66% and to be cost saving from both payer and societal perspectives [27]. Further, universal vaccination with a single vaccine dose in Mexico was projected to be cost saving from the perspective of the national payer system [39].

The effect of universal childhood varicella vaccination in Norway was modeled in a previous analysis [40]. This model, in which the first dose was given at 15 months and a second dose at 7 years, predicted a decline of 98.7% in varicella incidence within the first 5 years of program introduction [40]. This compares with a 79.8% reduction in the natural varicella incidence at 5 years with strategy A (V-MSD at 15 months and 7 years) in the present study. In the previous analysis, the predicted long-term (200 years) varicella incidence was about 20 cases per 100,000 person-years, reduced from 990 per 100,000 in the pre-vaccine era, representing a 97.8% reduction [40]. In the present study, universal varicella vaccination was projected to reduce the annual natural varicella incidence rate by 96.4% from 1,359 per 100,000 population to 49 per 100,000 under strategy A over 50 years.

Most (73%) of the annual healthcare costs of varicella virus infection in Norway are associated with herpes zoster, primarily due to hospitalization costs [12]. Our model showed that the incidence of herpes zoster is projected to decrease under all vaccination strategies, so that the healthcare costs associated with herpes zoster would also be expected to decrease. Hence, the costs associated with herpes zoster were not included in the base case of this model. However, we conducted a scenario analysis to assess the impact of accounting costs of HZ and found the costs of herpes zoster had a minimal impact (<10% change) on the cost-effectiveness of vaccine program.

As discussed above, the present model incorporated an exogenous boosting effect. Models of universal varicella vaccination that include exogenous boosting generally predict a transient increase in herpes zoster in adults [13, 16, 22, 30, 41–43]. The previous model of universal childhood varicella vaccination in Norway also included exogenous boosting, which predicted a substantial increase in herpes zoster incidence, with a peak approximately 50 years after vaccination that was 2.6 times higher than the pre-vaccine level [40]. However, the effects of universal varicella vaccination on herpes zoster incidence are strongly dependent on the hypothesized boosting intensity: the smaller the intensity, the smaller the increase in herpes zoster incidence. Similarly, other investigators have reported that the projected transient increases in herpes zoster are sensitive to a number of parameters whose values are uncertain [41].

In observational studies, the relationship between circulating varicella virus and herpes zoster epidemiology is unclear. A review of studies of different types was equivocal, in that the magnitude of any exogenous boosting effect was indeterminate [44]. More recently, the impact of varicella vaccination on the incidence of herpes zoster was inferred from observation of herpes zoster incidence before and after the introduction of childhood varicella vaccination in the United States [45]. Annual rates of change in herpes zoster incidence were determined in an interrupted time series regression analysis, for the periods of 1991–1995, 1996–2006, and 2007–2016, corresponding to the pre-vaccination, one-dose vaccination, and two-dose vaccination periods, respectively. The transient increase in herpes zoster incidence predicted by the exogenous boosting hypothesis was not observed [45].

From the perspectives of both society and the healthcare system, strategies with two doses of V-MSD (strategies A and E) were the most effective in reducing costs and making gains in quality of life. The cost of a caregiver’s lost work time had the greatest effect on costs from the societal perspective, though varying this cost by ± 20% resulted in only a ± 5.0% change in the ICER with strategy A. Varying vaccine price by ± 20% resulted in a ± 2.4% change in the ICER for strategy A from the societal perspective.

This analysis is subject to several limitations. The modeled vaccine administrations at 15 months, 7, and 11 years match Norway’s existing childhood vaccination program (MMR at 15 months and 11 years, and DTP-IPV at 7 years). We modeled a strategy that included a vaccination at 18 months (strategies E and F). However, there is no existing immunization visit at 18 months, which was the modelled timepoint for the second vaccine dose in strategies E and F. Any costs associated with this currently unscheduled visit were not included in the model, thus, the actual costs may be higher than estimated in the present study. Due to a lack of country-specific data for caregiver costs, the model employs published estimates from other countries. This is a conservative estimate of ICERs as the analysis utilizes list price for vaccines and not tender price. There are several other potential limitations to the modeling approach taken [13]. The model employed a static population size and age distribution, whereas the population of Norway is expected both to increase and to age over the coming decades. Other demographic changes were not captured by the model, including changes in fertility trends and changes in social contact patterns.

## Conclusions

In conclusion, universal childhood varicella vaccination with a two-dose schedule is projected to lead to substantial reductions in varicella incidence and mortality in Norway, with associated reductions in healthcare use and in direct and indirect costs. All modeled two-dose varicella vaccination strategies are cost saving, but the most cost-effective strategies are with V-MSD at both the first and second doses, with the second dose administered at either 18 months or 7 years [11]. Policymakers should consider universal varicella vaccination to reduce varicella disease and the caregiver and economic burdens in Norway.

## Supporting information

S1 Appendix Model details. (DOCX)

S2 Appendix Sensitivity analyses results for discounting, time horizon and HZ costs scenarios. (DOCX)

S3 Appendix Minimal data sets. (ZIP)
