# Supplementary material for: Clinical and economic impact of universal varicella vaccination in Norway: A modeling study
Source: PLoS One. 2021 Jul 8;16(7):e0254080. doi: 10.1371/journal.pone.0254080 (PMC8266049; doi:10.1371/journal.pone.0254080)
Supplement: S1 Appendix — (DOCX) [file pone.0254080.s001.docx]

S1 Appendix. Model details

Table 1. Vaccine characteristics for modeled vaccine formulations

| **Property** | **Definition** | **Dose** | **MSD varicella-containing vaccines** | **GSK varicella-containing vaccines** | **Source** |
| --- | --- | --- | --- | --- | --- |
| Failure | Percent of individuals for which vaccine fails to provide any protection | 1 & 2 | 4% | 5% | van Hoek et al. 2011 |
| Take (among the individuals who did not fail remaining 96% MSD and 95% GSK) | % of individuals permanently protected from breakthrough varicella with 1^st^ dose | 1^st^ | 90.3%  (87.8%-92.9%) | 61.7%  (58.1%-65.2%) | Model fit to clinical trial data |
|  | % of individuals unprotected or temporarily protected with 1^st^ dose that get permanently protected with 2^nd^ dose | 2^nd^ | 69.0%  (52.3%-85.7%) | 85.9%  (82.5%-89.3%) |  |
|  | Total performance (permanent protection) of 1^st^ and 2^nd^ dose | 1&2 | 97.0% (95.2%-98.8%) | 94.6% (93.2%-96.0%) |  |
| Duration of temporary protection for each vaccine | Temporarily protected individuals become susceptible to breakthrough varicella after vaccination | 1^st^ | 1.21y  (0.77y-2.84y) | 0.91y  (0.72y-1.23y) |  |
|  |  | 2^nd^ |  |  |  |

Table 2. Base model parameters

| **Symbol** | **Description** | | **Value** | **Source** |
| --- | --- | --- | --- | --- |
| **Disease-specific parameters** | | | | |
| $1/\omega_{m}$ | Average period of passive immunity | | 6 months | Gershon 1976 |
| $1/{\epsilon_{n}}, 1/{\epsilon_{vb}}$ | Average latent period (natural, breakthrough varicella) | | 14 days | Gordon 1929 |
| $1/{\gamma_{n}}$ | Average infectious period for natural varicella | | 7 days | Izurieta 1997 |
| $1/{\gamma_{bv}}$ | Average infectious period for breakthrough varicella | | 4.5 days | Izurieta 1997 |
| $1/\eta_{n},1/\eta_{vv},1/\eta_{vb}$ | Average length of HZ case (wild-type, vaccine-type, wild-type after breakthrough varicella) | | 28 days | Garnett 1992 |
| $1/{\delta_{n}}, 1/{\delta_{vv}}, 1/{\delta_{vb}}$ | Average duration of high HZ immunity | | 79.7 years | Poletti 2013 |
| $\rho_{v}$ | Relative infectivity of breakthrough varicella infection | | 50% | Coudeville 2005 |
| $\rho_{z}$ | Relative infectivity of HZ infection | | 0.07 | Schuette 1999 |
| $\zeta_{n},\zeta_{vv},\zeta_{vb}$ | Percentage of contacts leading to exogenous boosting | | 100% | Brisson Edmunds 2000 |
| $\xi_{n},\xi_{vv},\xi_{vb}$ | Rate of endogenous boosting | | 0 | (Assumed) |
| χ | Reactivation rate factor on vaccine arms | | 1/6 | Poletti 2013 |
| Fertility | Births by age of mother  Age (years) | | Births/1,000 | Spackova et al. 2010; Statistics Norway's Information Centre |
|  | 15 - 20 | | 5.0 |  |
|  | 20 -25 | | 64.6 |  |
|  | 25 – 30 | | 193.8 |  |
|  | 30 - 35 | | 227.2 |  |
|  | 35 – 40 | | 109.7 |  |
|  | 40 – 45 | | 21.9 |  |
|  | 45 - 50 | | 1.6 |  |
| *cfr_z_* | HZ case fatality ratio by age | | Cases / 100,000 | Edmunds et al 2001; Haugnes 2017 |
|  | <5 | | 0 |  |
|  | 5 - 15 | | 0 |  |
|  | 15 – 80 | | 11 |  |
|  | >80 | | 11 |  |
| *cfr_n_, cfr_vb_* | Varicella case fatality ratio | | Cases / 100,000 | Zhou et al 2008 |
|  | <1 | | 3.7 |  |
|  | 1-5 | | 0.8 |  |
|  | 5-10 | | 1.0 |  |
|  | 10-15 | | 1.6 |  |
|  | 15-20 | | 5.9 |  |
|  | 20-40 | | 21.3 |  |
|  | 40-45 | | 25.0 |  |
|  | 45-65 | | 120.0 |  |
|  | >65 | | 1170.0 |  |
| *p_phn_* | Age-specific probability of HZ developing into PHN | | % | Brisson & Edmunds 2003 |
|  | <5 | | 0 |  |
|  | 5 - 15 | | 1 |  |
|  | 15 – 45 | | 4 |  |
|  | 45 - 65 | | 11 |  |
|  | >65 | | 31 |  |
| *d_phn_* | Average duration of PHN (days) | | 511 | Brisson & Edmunds 2003 |
| **Varicella vaccine-related parameters (default vaccine, Varivax®)** | | | | |
| *F*=(1-*P*) | Vaccine failure | | 4% | van Hoek 2011 |
| *T_1_* | Varicella vaccine 1^st^ dose take | | 100% | van Hoek 2011 |
| *T_2_* | Varicella vaccine 2^nd^ dose take | | 100% | van Hoek 2011 |
| $1/{\omega_{v1}}$ | Average duration of 1^st^ dose varicella vaccine immunity to varicella | | 25 years | van Hoek 2011 |
| $1/{\omega_{v2}}$ | Average duration of 2^nd^ dose varicella vaccine immunity to varicella | | 77 years | van Hoek 2011 |
| **Varicella vaccine-related HZ protection parameters (assumed same for all varicella vaccines)** | | | | |
| $1/{\pi_{1}},1/{\pi_{2}},$ | | Average duration of 1^st^, 2^nd^ dose varicella vaccine induced high HZ immunity | same as vaccine duration of protection | (Assumed) |
| 1- *b_v_*_1_, 1- *b_v_*_2_ | | Varicella vaccine 1^st^, 2^nd^ dose degree of protection | 0 | (Assumed) |
| *k_v_*_1,_ *k_v_*_2_ | | Percentage of 1^st^ /2^nd^ dose protected who are boosted to full HZ protection from contact with varicella | 100% | van Hoek 2011 |

HZ, herpes zoster; PHN, postherpetic neuralgia; VZV, varicella zoster virus

Table 3. Health state utility values used in the model ^A^

| **Health state** | **Utility by age (years)** | | | | | |
| --- | --- | --- | --- | --- | --- | --- |
| Healthy (q_h_) | Ages | <40 | 40 - 60 | 60 - 70 | 70 - 80 | ≥80 |
|  | Utility | 1 | 0.901 | 0.871 | 0.833 | 0.729 |
| Natural varicella (q_n_) | Ages | <15 | ≥15 |  |  |  |
|  | Utility | 0.79 | 0.74 |  |  |  |
| Breakthrough varicella (q_vv_) | Ages | <15 | ≥15 |  |  |  |
|  | Utility | 0.92 | 0.92 |  |  |  |
| Uncomplicated HZ (q_zv_) | Ages | All |  |  |  |  |
|  | Utility | 0.87 |  |  |  |  |
| PHN zoster (q_phn_) | Ages | All |  |  |  |  |
|  | Utility | 0.671 |  |  |  |  |

HZ, herpes zoster; PHN, postherpetic neuralgia

^A^ Values derived from Littlewood 2015.

Table 4. Mean direct and indirect treatment costs, by age group for varicella and herpes zoster cases for Norway ^A^

|  |  | **Age group (years) ^B^** | |
| --- | --- | --- | --- |
|  | **All ages** | **0-14** | **≥15** |
| Varicella-related outpatient ^C^ | - | 131.20 | 262.39 |
| Varicella-related inpatient ^C^ | - | 4,141.06 | 8,282.13 |
| Uncomplicated HZ ^C^ | 292.14 | - | - |
| HZ postherpetic neuralgia ^C^ | 24,675.11 | - | - |

HZ, herpes zoster

^A^ Costs reported in 2020 NOK.

^B^ Age group boundaries are closed on the left (boundary is included in the group) and open on the right (boundary is not included in the group).

^C^ Values derived from Haugnes et al 2019.

Table 5. Mean indirect treatment costs for varicella cases for Norway ^A^

|  | **Workdays lost** | | **Cost ^C^** |
| --- | --- | --- | --- |
|  | **Outpatient ^B^** | **Inpatient ^B^** |  |
| Natural varicella | 5.0 | 5.0 | 2,347.42 |
| Breakthrough varicella | 2.5 | 2.5 |  |
| Herpes zoster | 5.84 ^D^ | |  |

^A^ Costs reported in 2020 NOK.

^B^ Values derived from Norwegian Institute of Public Health 2019.

^C^ Mean cost per workday lost from (Statistics Norway's Information Centre).

^D^ Value derived from (Drolet 2012). 43.8 hours lost / 7.5 hours per day = 5.84 days lost for all individuals >15 years.

Table 6. Health outcomes base values

| Parameter | Default Value | | References and Assumptions |
| --- | --- | --- | --- |
|  | < 15 Years | ≥ 15 years |  |
| Natural varicella case |  |  |  |
| Duration (days) | 7.0 | 7.0 | Brisson and Edmunds 2003 |
| Utility | 0.79 | 0.74 | Brisson and Edmunds 2003 |
| **Calculated QALY loss per case** | **0.004** | **0.005** | (Calculated value) |
| Breakthrough varicella case |  |  |  |
| Duration (days) | 4.5 | 4.5 | Brisson and Edmunds 2003 |
| Utility | 0.92 | 0.92 | Brisson and Edmunds 2003 |
| **Calculated QALY loss per case** | **0.001** | **0.001** | (Calculated value) |
| Herpes zoster |  |  |  |
| Duration (days) |  |  |  |
| Acute HZ case | 14.0 | | Brisson and Edmunds 2003 |
| HZ case with PHN | 511.0 | | Brisson and Edmunds 2003 |
| Utility by pain level |  |  |  |
| None | 1.000 | | Brisson and Edmunds 2003 |
| Mild | 0.730 | | Brisson and Edmunds 2003 |
| Severe | 0.470 | | Brisson and Edmunds 2003 |
| Percentage of cases that experience pain | |  |  |
| Acute HZ case | 82.0% | | Brisson and Edmunds 2003 |
| HZ case with PHN | 100.0% | | Brisson and Edmunds 2003 |
| Distribution of pain |  |  |  |
| Mild | 77.3% | | Brisson and Edmunds 2003 |
| Severe | 22.7% | | Brisson and Edmunds 2003 |
| **Calculated QALY loss per case** |  |  |  |
| Acute HZ case | **0.010** | | (Calculated value) |
| HZ case with PHN | **0.461** | | (Calculated value) |

HZ, herpes zoster; PHN, postherpetic neuralgia; QALY, quality-adjusted life year

**References**

Brisson M, Edmunds WJ, Gay NJ, Law B, De Serres G. Modelling the impact of immunization on the epidemiology of varicella zoster virus. Epidemiol Infect. 2000; 125:651–669. https://doi.org/10.1017/s0950268800004714 PMID: 11218215.

Brisson M, Edmunds WJ, Gay NJ, Law B, De Serres G. Analysis of varicella vaccine breakthrough rates: implications for the effectiveness of immunisation programmes. Vaccine. 2000; 18:2775–2778. https://doi.org/10.1016/s0264-410x(00)00100-6 PMID: 10812218.

Brisson M, Edmunds WJ. Varicella vaccination in England and Wales: cost-utility analysis. Arch Dis Child. 2003; 88:862-869.

Coudeville L, Brunot A, Szucs TD, Dervaux B. The economic value of childhood varicella vaccination in France and Germany. Value Health. 2005; 8:209-222. doi: 10.1111/j.1524-4733.2005.04005.x.

Drolet M, Levin MJ, Schmader KE, Johnson R, Oxman MN, Patrick D, et al. Employment related productivity loss associated with herpes zoster and postherpetic neuralgia: a 6-month prospective study. Vaccine. 2012;30(12):2047-50.

Edmunds WJ, Brisson M, Rose JD. The epidemiology of herpes zoster and potential cost-effectiveness of vaccination in England and Wales. Vaccine. 2001; 19:3076–3090. <https://doi.org/10.1016/s0264->410x(01)00044-5 PMID: 11312002.

Garnett GP, Grenfell BT. The epidemiology of varicella-zoster virus infections: a mathematical model. Epidemiol Infect. 1992; 108:495-511.

Gershon AA, Raker R, Steinberg S, Topf-Olstein B, Drusin LM. Antibody to Varicella-Zoster virus in parturient women and their offspring during the first year of life. Pediatrics. 1976; 58:692-696.

Gordon JE, Meader FM. The period of infectivity and serum prevention of chickenpox. JAMA. 1929; 93:2013-2015. doi: 10.1001/jama.1929.02710260001001.

Haugnes H. 2017. The financial burden of the varicella zoster virus in Norway and a cost-utility analysis of preventive action. Thesis submitted as a part of the Master of Philosophy Degree in Health Economics, Policy, and Management UNIVERSITY OF OSLO, Faculty of Medicine Department of Health Management and Health Economics, May 2017.

Haugnes H, Flem E, Wisløff T. Healthcare costs associated with varicella and herpes zoster in Norway. Vaccine. 2019;37(29):3779-3784.

Izurieta HS, Strebel PM, Blake PA. Postlicensure effectiveness of varicella vaccine during an outbreak in a child care center. JAMA. 1997; 278:1495-1499.

Littlewood KJ, Ouwens MJ, Sauboin C, Tehard B, Alain S, Denis F. Cost-effectiveness of routine varicella vaccination using the measles, mumps, rubella and varicella vaccine in France: an economic analysis based on a dynamic transmission model for varicella and herpes zoster. Clin Ther. 2015; 37:830–841 e837. https://doi.org/10.1016/j.clinthera.2015.01.006 PMID: 25721380.

Norwegian Institute of Public Health. Vaccination of adults and risk groups. 2018. Available at: https://www.nsf.no/Content/4136272/cache=1541764950000/Utredning+vaksinasjonsprogram+for+voksne+og+risikogrupper.pdf. Accessed December 7, 2020.

Poletti P, Melegaro A, Ajelli M, Del Fava E, Guzzetta G, Faustini L, et al. Perspectives on the impact of varicella immunization on herpes zoster. A model-based evaluation from three European countries. PLoS One. 2013; 8:e60732. https://doi.org/10.1371/journal.pone.0060732 PMID: 23613740.

Spackova M, Wiese-Posselt M, Dehnert M, Matysiak-Klose D, Heininger U, Siedler A. Comparative varicella vaccine effectiveness during outbreaks in day-care centres. Vaccine. 2010; 28:686–691. https://doi.org/10.1016/j.vaccine.2009.10.086 PMID: 19874924.

Schuette MC, Hethcote HW. Modeling the effects of varicella vaccination programs on the incidence of chickenpox and shingles. Bull Math Biol. 1999; 61:1031–1064. PMID: 17879870.

Statistics Norway. 07902: Life tables, by sex and age 1966 – 2019. 2019. Available at: https://www.ssb.no/en/statbank/table/07902/. Accessed July 15, 2020.

Statistics Norway. 11652: Employees, jobs, and earnings, by place of work, sex, and age. 2020a. Available at: https://www.ssb.no/en/statbank/table/11652. Accessed July 15, 2020.

Statistics Norway. 11656: Employees and jobs, by sex, age, and industry division. 2020b. Available at https://www.ssb.no/en/statbank/table/11656. Accessed July 15, 2020.

van Hoek AJ, Melegaro A, Zagheni E, Edmunds WJ, Gay N. Modelling the impact of a combined varicella and zoster vaccination programme on the epidemiology of varicella zoster virus in England. Vaccine. 2011; 29:2411–2420. https://doi.org/10.1016/j.vaccine.2011.01.037 PMID: 21277405.

Zhou et al. 2008. An Economic Analysis of the Universal Varicella Vaccination Program in the United States. JID 197(S2): S156-S164.
