# Supplementary material for: Clinical and economic impact of universal varicella vaccination in Norway: A modeling study
Source: PLoS One. 2021 Jul 8;16(7):e0254080. doi: 10.1371/journal.pone.0254080 (PMC8266049; doi:10.1371/journal.pone.0254080)
Supplement: S2 Appendix — (DOCX) [file pone.0254080.s002.docx]

S2 Appendix. Sensitivity analyses results for discounting, time horizon and HZ costs scenarios

Table 1. ICER (incremental cost-effectiveness ratio) values from the societal perspective, for 3% and 4% discounting and time horizons of 25, 50, and 100 years ^A^

| Time horizon | Strategy |  | ICER | |  | % difference ^B^ |
| --- | --- | --- | --- | --- | --- | --- |
|  |  |  | 3% discounting | 4% discounting |  |  |
| **25 years** |  |  |  |  |  |  |
|  | A |  | -1,408,634 | -1,409,823 |  | 0.08% |
|  | B |  | -1,303,667 | -1,303,574 |  | -0.01% |
|  | C |  | -1,364,708 | -1,369,170 |  | 0.33% |
|  | D |  | -1,193,664 | -1,196,991 |  | 0.28% |
|  | E |  | -1,416,246 | -1,416,079 |  | -0.01% |
|  | F |  | -1,361,194 | -1,361,374 |  | 0.01% |
| **50 years** |  |  |  |  |  |  |
|  | A |  | -1,387,190 | -1,391,457 |  | 0.31% |
|  | B |  | -1,296,066 | -1,297,364 |  | 0.10% |
|  | C |  | -1,341,954 | -1,348,558 |  | 0.49% |
|  | D |  | -1,213,135 | -1,213,028 |  | -0.01% |
|  | E |  | -1,392,018 | -1,395,829 |  | 0.27% |
|  | F |  | -1,331,833 | -1,336,461 |  | 0.35% |
| 100 years |  |  |  |  |  |  |
|  | A |  | -1,366,335 | -1,377,019 |  | 0.78% |
|  | B |  | -1,284,188 | -1,289,370 |  | 0.40% |
|  | C |  | -1,321,741 | -1,334,153 |  | 0.94% |
|  | D |  | -1,211,547 | -1,212,193 |  | 0.05% |
|  | E |  | -1,370,112 | -1,380,770 |  | 0.78% |
|  | F |  | -1,312,065 | -1,322,636 |  | 0.81% |

^A^ The base case is a time horizon of 50 years and 3% discounting rate. Costs are in NOK.

^B^ Percent difference in incremental cost-effectiveness ratio between 4% and 3% discounting rate.

Table 2. Cost-effectiveness of universal varicella vaccination in Norway from the perspective of the health care system after including costs of herpes zoster ^A^

| Vaccination strategy | Cost savings in NOK ^B^ | QALYs gained | ICER ^B^ | Percent change ^C^ |
| --- | --- | --- | --- | --- |
| A | (126.44) | 0.00138 | (91,731) | 3.5% |
| B | (96.89) | 0.00135 | (71,866) | 2.4% |
| C | (85.75) | 0.00137 | (62,808) | 1.1% |
| D | (47.07) | 0.00132 | (35,558) | -5.1% |
| E | (119.94) | 0.00139 | (86,080) | 3.2% |
| F | (102.85) | 0.00137 | (74,804) | 2.6% |

QALY, quality-adjusted life years; ICER, incremental cost-effectiveness ratio.

^A^ The time horizon is 50 years.

^B^ Costs in NOK 2020. The parentheses indicate negative values which represent cost savings.

^C^ Percent change in incremental cost-effectiveness ratio from base case results that did not include herpes zoster costs in the cost-effectiveness analysis.

Table 3. Cost-effectiveness of universal varicella vaccination in Norway from the perspective of the societal perspective after including costs of herpes zoster ^A^

| Vaccination strategy | Cost savings in NOK ^B^ | QALYs gained | ICER ^B^ | Percent change ^C^ |
| --- | --- | --- | --- | --- |
| A | (1,799.12) | 0.00138 | (1,305,231) | 5.9% |
| B | (1,638.09) | 0.00135 | (1,214,965) | 6.3% |
| C | (1,728.15) | 0.00137 | (1,265,773) | 5.7% |
| D | (1,508.73) | 0.00132 | (1,139.664) | 6.1% |
| E | (1,821.41) | 0.00139 | (1,307,205) | 6.1% |
| F | (1,714.05) | 0.00137 | (1,246,639) | 6.4% |

QALY, quality-adjusted life years; ICER, incremental cost-effectiveness ratio.

^A^ The time horizon is 50 years.

^B^ Costs in NOK 2020. The parentheses indicate negative values which represent cost savings.

^C^ Percent change in incremental cost-effectiveness ratio from base case results that did not include herpes zoster costs in the cost-effectiveness analysis.
